# High-Performance Uncooled Infrared Photodetection Based on van der Waals Metallic FePd_2_Te_2_ Nanoflakes

**DOI:** 10.3390/nano16201289

**Published:** 2026-10-11

**Authors:** Kang Zhang, Yeqi Shen, Wenke Xu, Tao Han, Yubing Tu, Suofu Wang, Mingsheng Long

**Affiliations:** 1 State Key Laboratory of Opto-Electronic Information Acquisition and Protection Technology, Institutes of Physical Science and Information Technology, Anhui University, Hefei 230601, China; 2 School of Energy, Power and Safety, Anhui University of Applied Technology, Hefei 230011, China

**Keywords:** negative photoconductivity, photobolometric effect, FePd_2_Te_2_, photodetectors

## Abstract

Uncooled infrared photodetectors that combine high sensitivity, low power consumption, and broad spectral coverage remain highly desirable. However, they are still hindered by the complex epitaxial processes and cooling requirements of mainstream semiconductor technologies. Here, we demonstrate a high-performance infrared photodetector based on FePd_2_Te_2_ nanoflakes, a van der Waals metallic material that enables efficient photothermal conversion. The device operates at room temperature under a bias of 0.1 V and exhibits a broadband photoresponse spanning from the visible to the long-wave infrared. Across the measured infrared range, a wavelength-independent responsivity (*R*) above 50 A/W, a noise equivalent power (*NEP*) below 2.83 × 10^−16^ W/Hz^1/2^, and a specific detectivity (D*) exceeding 3.86 × 10^12^ cm Hz^1/2^/W are achieved. The photoresponse appears to arise from the photobolometric effect, in which the temperature rise at the contact interface is considered to modulate the contact resistance and thereby amplify the photocurrent. These results establish FePd_2_Te_2_ as a promising platform for room-temperature, low-power, and highly sensitive infrared detection.

## 1. Introduction

Infrared photodetection is indispensable in fields such as optical fiber communications, target recognition, remote sensing, environmental monitoring, and military surveillance [1,2]. With the rapid advancement of the Internet of Things and intelligent sensing technologies, the demand for high-performance, low-cost infrared photodetectors capable of room-temperature operation has become increasingly urgent. However, current mainstream infrared detection technologies still rely heavily on single-crystalline germanium (Ge) and III–V semiconductor materials such as indium gallium arsenide (InGaAs), indium antimonide (InSb), and mercury cadmium telluride (HgCdTe) [3,4,5]. Although systems based on these materials have achieved a high level of detection performance, their fabrication processes are extremely complex, typically relying on high-precision, high-cost epitaxial techniques such as molecular beam epitaxy, metal–organic chemical vapor deposition, and liquid phase epitaxy [6], which not only entail enormous equipment investment but also suffer from poor compatibility with mainstream complementary metal-oxide-semiconductor processes, making large-scale, low-cost monolithic integration difficult to achieve [7,8,9].

Since the discovery of graphene, two-dimensional (2D) materials have attracted extensive attention for their unique electronic and optoelectronic properties [10,11,12,13]. Infrared photodetectors based on 2D materials have demonstrated remarkable progress over the past few decades. For instance, narrow bandgap 2D semiconductors BP and b-AsP have demonstrated excellent infrared photoresponse [14,15]. Meanwhile, transition metal dichalcogenides such as PtS_2_ [16,17], PtSe_2_ [18,19], PtTe_2_ [20,21], PdTe_2_ [22,23], and PdSe_2_ [24,25], have garnered extensive attention in infrared photodetection owing to their suitable bandgaps ranging from the visible to the long-wave infrared (LWIR) spectral range, strong light absorption, and excellent environmental stability. However, the environmental instability of BP and b-AsP poses an enormous challenge to practical device applications. In addition, the relatively wide bandgap, together with the thermal noise arising from high dark current, constrains transition metal dichalcogenides to limited photoresponsivity and low quantum efficiency in the mid-wave infrared (MWIR) and LWIR bands [19].

Unlike semiconductor photodiodes, in which electron–hole pair separation is limited by the photon energy, thermal photodetectors absorb radiation as heat and transduce the resulting temperature rise into an electrical signal [26]. This mechanism enables broadband detection from the visible to the far-infrared. For example, VO_2_ with the photobolometric effect is one of the most common thermistor materials, and La_2/3_Ca_1/3_MnO_3_ films with a large temperature coefficient of resistance show high sensitivity induced by the photothermal effect [27]. Moreover, the photobolometric effect enables Fe_3_O_4_ nanoflakes to achieve an ultrahigh photoresponsivity *R* of 561.2 A W^−1^ at 10.6 µm [28], while the 2D α-Fe_2_O_3_ photodetector delivers a high specific detectivity D* of 3.28 × 10^9^ cm Hz^1/2^ W^−1^ and a very low *NEP* of 8.94 × 10^−14^ W Hz^−1/2^ under 10.6 µm laser illumination [29]. However, these detectors face challenges in material fabrication, and the realization of superior infrared photoresponse largely relies on high external bias voltages, while their sensitivity still requires further improvement, all of which hinder the development of low-power-consumption and high-sensitivity infrared optoelectronic devices.

Herein, we report a high-performance uncooled infrared photodetector based on FePd_2_Te_2_ nanoflakes. The device demonstrates broadband photodetection capability ranging from visible light to long-wavelength infrared. At an external bias of 0.1 V, it exhibits excellent infrared detection capability, with a responsivity above 50 A/W, an *NEP* below 2.83 × 10^−16^ W/Hz^1/2^ and a D* exceeding 3.86 × 10^12^ cm Hz^1/2^/W across the entire measured infrared range, indicating high sensitivity for infrared detection. This work provides a promising route for the development of room-temperature, low-power-consumption, and high-sensitivity infrared photodetectors.

## 2. Materials and Methods

FePd_2_Te_2_ single crystals were grown by melting stoichiometric elements in a molar ratio of 1:2:2 (Fe:Pd:Te). X-ray diffraction (XRD) patterns were collected on a Rigaku SmartLab (Rigaku Corp., Tokyo, Japan) with Cu-Kα_1_ radiation at room temperature. The thickness of the FePd_2_Te_2_ nanoflake was characterized using an atomic force microscope (AFM, Oxford, Santa Barbara, CA, USA). Energy-dispersive X-ray spectroscopy (EDS) elemental analysis was carried out using a Zeiss Crossbeam 550 L system (Carl Zeiss Microscopy GmbH, Oberkochen, Germany).

The FePd_2_Te_2_ nanoflakes were obtained via standard mechanical exfoliation. The dry transfer method was then employed to place the flake onto a prefabricated bottom electrode (Cr/Au, ~5/30 nm) designed to enhance laser reflection. The substrate consists of a heavily doped Si substrate (0.5 mm, orientation: ⟨100⟩, resistivity 0.01–0.05 Ω cm, Hefei Kejing Material Technology Co., Ltd., Hefei, China) covered by 300 nm SiO_2_. The top metal contacts, consisting of Cr/Au (typically 10 nm Cr and 80 nm Au), were defined by standard electron beam lithography (EBL) followed by thermal evaporation. A dual-channel digital source meter (Keithley 2636B, Keithley Instruments, Cleveland, OH, USA) was used for both electrical characterization and photoresponse measurements. For visible-light excitation at 637 nm, the laser was focused onto the device through a 20× objective lens. MWIR performance was evaluated using a wavelength-tunable multi-channel laser covering 2.5–4.2 μm, with a beam diameter of approximately 4.4 mm. Long-wavelength infrared (LWIR) measurements at 10.6 μm were carried out with a CO_2_ laser source. All measurements were conducted in ambient air at room temperature.

## 3. Result and Discussion

**Figure 1:**
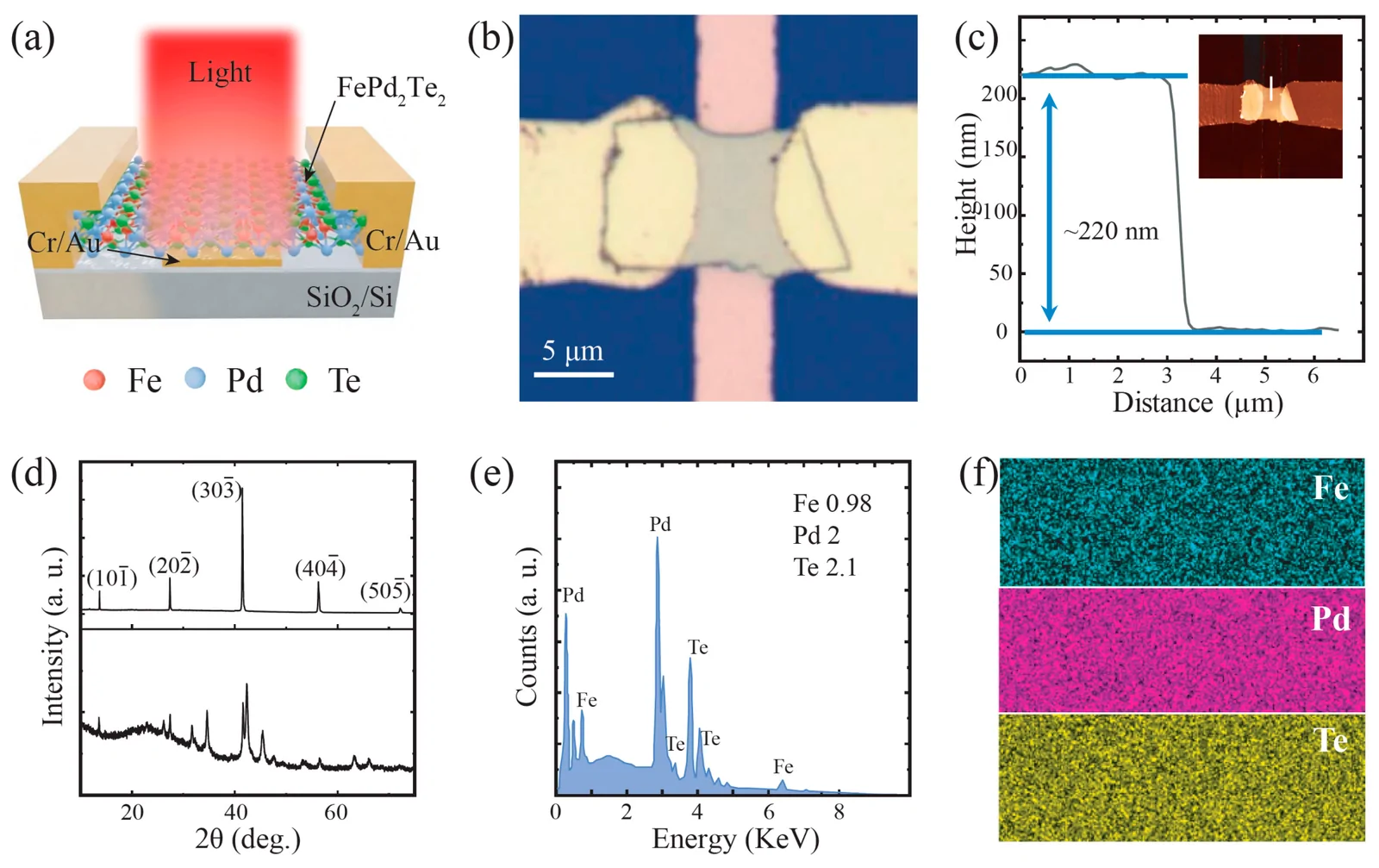
Characterizations of the FePd_2_Te_2_ single crystal and device configuration. (**a**) Schematic illustration of the FePd_2_Te_2_ device. (**b**) Optical image of the FePd_2_Te_2_ nanoflake. (**c**) Step-height profile of the FePd_2_Te_2_ flake. The inset shows the AFM image of the device. (**d**) XRD patterns collected by single-crystal XRD (upper panel) and powder XRD (lower panel) of crushed single crystals. (**e**) EDS spectrum of the FePd_2_Te_2_ nanoflake. (**f**) Elemental mapping of the measured FePd_2_Te_2_ nanoflake.

A schematic illustration of the FePd_2_Te_2_ detector is presented in Figure 1a, depicting its layered crystal structure with characteristic atomic arrangement. Figure 1b displays the optical image of the fabricated detector, revealing a well-defined flake morphology with a visually smooth and flat surface (height variation of ~3 nm; Supplementary Figure S1). AFM was employed to characterize the FePd_2_Te_2_ detector, and the height profile measurement confirmed a nanoflake thickness of approximately 220 nm, as shown in Figure 1c. The clean topography observed by AFM further attests to the uniformity and flatness of the exfoliated flake. To verify the crystallographic orientation, both single-crystal and powder XRD measurements were performed, displayed in Figure 1d. The XRD patterns exhibit a series of prominent [10$\bar{1}$] diffraction peaks, indicating that the FePd_2_Te_2_ nanoflake grows precisely along the [10$\bar{1}$] crystallographic direction, consistent with the natural cleavage plane of this van der Waals material [30]. EDS was also conducted to examine the elemental composition and distribution. The EDS spectrum confirms the presence of Fe, Pd, and Te elements with a stoichiometric ratio consistent with the nominal composition of FePd_2_Te_2_, as shown in Figure 1e,f. Furthermore, the EDS elemental mapping reveals a regular flake morphology and a highly uniform spatial distribution of Fe, Pd, and Te elements across the entire nanoflake, with no detectable impurity segregation. Collectively, these results demonstrate that a high-quality FePd_2_Te_2_ single crystal was obtained, providing a reliable platform for device fabrication and subsequent optoelectronic measurements.

**Figure 2:**
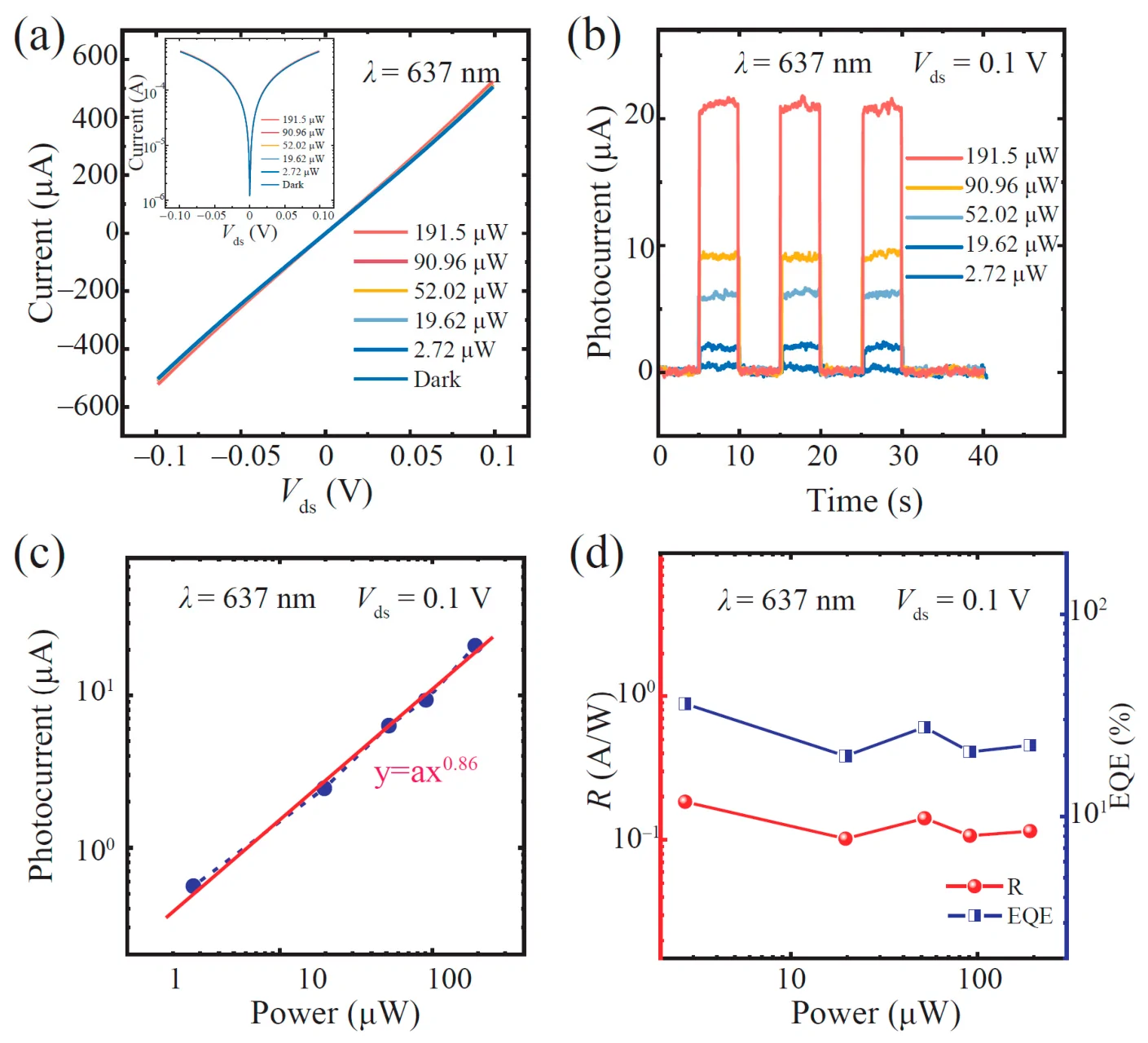
Optoelectronic response of the FePd_2_Te_2_ photodetector under 637 nm illumination. (**a**) *I*-*V* characteristics of the FePd_2_Te_2_ device. The inset shows the semilogarithmic plot of the *I*-*V* curves. (**b**) Temporal photoresponse under various light power intensities. (**c**) Photocurrent as a function of incident power at 0.1 V. (**d**) Extracted *R* and EQE of the device as functions of incident power at 0.1 V.

We then systematically investigated the photoresponse of the FePd_2_Te_2_ device. Figure 2a shows the current–voltage (*I*-*V*) curves measured in the dark and under various illumination powers of a 637 nm laser. Within the applied bias range of ±0.1 V, the photocurrent at an incident power of 191.5 μW increased only weakly relative to the dark condition. Nevertheless, the time-resolved measurements in Figure 2b show that the current rose and fell periodically with the switching of the illumination, indicating a pronounced positive photoresponse. This behavior is distinctly different from that of FePd_2_Te_2_ devices fabricated on bottom electrodes [31]. The light-power dependence of the photocurrent was fitted using a power–law relationship *I*_ph_∝*P*^α^. The photocurrent was calculated as *I*_ph_ = *I*_L_ − *I*_D_, where *I*_L_ and *I*_D_ are the currents under illuminated and dark conditions, respectively. Here *P* is the effective incident power corrected by the device-to-beam area ratio. The illumination conditions and optical-power calculation are summarized in Supplementary Table S1. The fitting yields α = 0.86 at 637 nm, indicating a sublinear response (Figure 2c). To quantitatively evaluate the device performance, we calculated the responsivity *R*, defined as the ratio of the photocurrent (*I*_ph_) to the incident light power (*P*_I_): *R* = *I*_ph_/*P*_I_. The photocurrent was obtained from *I*_ph_ = *I*_L_ − *I*_D_, where *I*_L_ and *I*_D_ represent the currents with and without illumination, respectively. To assess the efficiency of photo-to-current conversion, we further calculated the external quantum efficiency (EQE), defined as the ratio of the number of conduction electrons contributing to the photocurrent to the number of incident photons. It can be expressed as EQE = *Rhc*/*eλ*, where *λ* is the wavelength of the incident light, *e* is the elementary charge, *c* is the speed of light, and *h* is the Planck constant. The extracted *R* and EQE as a function of incident light power at a bias of 0.1 V are presented in Figure 2d. Both *R* and EQE exhibit only a weak dependence on the incident power, yet they were significantly higher than those of the FePd_2_Te_2_ device on bottom electrodes [31].

Given the linear *I*-*V* characteristics and the metallic nature of FePd_2_Te_2_, photovoltaic and photogating effects cannot account for the positive response observed here. Compared with the bottom electrode configuration, a crucial difference lies in the contact quality: the transfer-based bottom electrode avoids Fermi-level pinning and enables high-quality contact, whereas the EBL process may introduce interfacial states and contact barriers, leading to a more complex photoresponse. Two possible scenarios may arise from the EBL defined contacts. (1) Photoconductive effect at the barrier interface: Under an applied bias, a local electric field is generated at these barrier regions. Upon illumination, photoexcited electron–hole pairs generated near the interface immediately experience this local electric field, enabling efficient separation and collection of photogenerated carriers. In this scenario, the photoconductive effect dominates over the relatively weak negative photoconductivity induced by the photobolometric effect, analogous to the case of few-layer graphene-based photodetectors [32]. (2) Contact-resistance-dominated photobolometric effect: FePd_2_Te_2_ is metallic with low intrinsic resistance, whereas the EBL contact introduces a relatively large interfacial contact resistance that likely exhibits a negative temperature coefficient. Upon illumination, the resulting temperature rise lowers the contact barrier height and thereby reduces the contact resistance. A similar behavior has been reported in the analogous 2D ferromagnetic metal Fe_3_GeTe_2_, where the photocurrent polarity follows the nonmonotonic resistance-temperature relationship of the conductive channel [33]. In this FePd_2_Te_2_ device, however, the dominant contribution is most likely to arise from the contact resistance rather than the bulk. Although the bulk resistance of FePd_2_Te_2_ increases with temperature [30], the reduction in contact resistance dominates the overall device resistance, yielding a net positive photoresponse.

**Figure 3:**
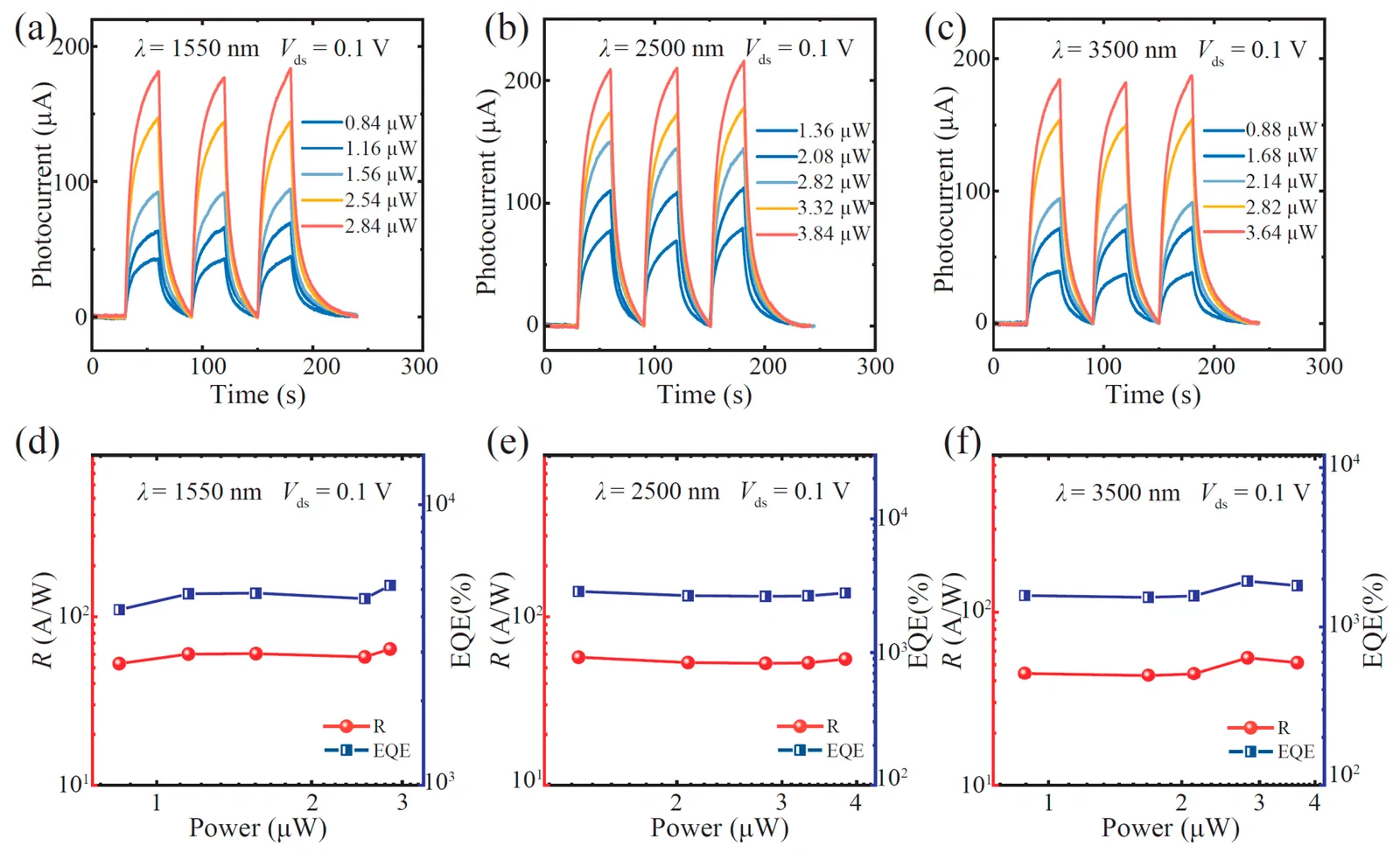
Optoelectronic response of the FePd_2_Te_2_ photodetector under 1550, 2500, and 3500 nm illumination. (**a**–**c**) Time-resolved photoresponse of the device under various incident powers at 1550, 2500, and 3500 nm, respectively. (**d**–**f**) Extracted *R* and EQE of the device under various incident powers at 1550, 2500, and 3500 nm, respectively.

To distinguish between these two scenarios, we further examined the wavelength-dependent photoresponse. Different from the photoresponse under visible light, the device exhibited a significantly more pronounced photoresponse under near-infrared (NIR) irradiation. The time-resolved photoresponse under 1550 nm illumination is shown in Figure 3a. The current variation is more substantial than that observed at 637 nm and increases progressively with incident power. It is noted that the infrared transients are obviously slower than the visible response, which is consistent with the thermal origin of the photoresponse. A related discussion on multi-relaxation behavior in 2D MoS_2_ phototransistors can be found in ref [34], although the metallic FePd_2_Te_2_ system and its photobolometric mechanism differ from the semiconductor MoS_2_ system. For the infrared measurements, the photoresponse was too slow to reach a steady-state plateau; therefore, the maximum photocurrent within the illumination pulse was used to estimate the responsivity, which may slightly underestimate the true steady-state value. *R* and EQE as a function of incident light power were also calculated at a bias of 0.1 V, as plotted in Figure 3d. Both *R* and EQE exhibited a power-independent behavior, consistent with the observations under 637nm illumination. However, the values obtained at 1550 nm are substantially higher. Specifically, *R* reached ~65 A/W and EQE reached ~5 × 10^3^%, representing a significant enhancement compared to the corresponding values of ~0.3 A/W and ~50% obtained at 637 nm. According to scenario (1), the stronger response at 1550 nm would require more efficient photocarrier generation. However, the photon energy of 1550 nm light is lower than that of 637 nm light, which is unfavorable for generating more electron–hole pairs. Moreover, the EQE exceeding 50 at 1550 nm cannot be explained by a simple photoconductive process or the photogating gain mechanism at the contact interface. The photoconductive effect is inefficient in a metallic channel without a band gap, while photogating requires a semiconducting channel with a low carrier concentration. Therefore, scenario (1) is unlikely to be responsible for the observed behavior. Instead, the wavelength dependence is most likely explained by scenario (2), based on the different absorption depths and the resulting heating efficiency at the contact interface. For 637 nm visible light, the absorption coefficient of FePd_2_Te_2_ is large and the penetration depth is extremely shallow [35]. Photothermal heating is thus confined to an ultrathin surface layer. This heated layer is in intimate contact with the substrate and the surrounding environment, allowing rapid heat dissipation through thermal conduction. Consequently, the temperature rise is small (weak response), while the heat is generated and dissipated quickly (fast response). This is fully consistent with the weak and fast response observed at 637 nm. For NIR light (1550 nm), the significantly increased penetration depth allows the photogenerated heat to be distributed uniformly throughout the material, forming a volumetric heat source. The thermal diffusion path from the interior of the material to the surface/substrate is longer, and the thermal diffusion time increases accordingly. In addition, the surrounding substrate and the underlying prefabricated electrode are also illuminated, which may further influence the heat dissipation pathway. These combined effects result in a larger temperature rise and a stronger, slower photoresponse.

We next examined the device performance in the MWIR-LWIR spectral range. The temporal photoresponse at 0.1 V under 2500 nm and 3500 nm illumination are presented in Figure 3b,c, respectively. Under 2500 nm excitation, a photocurrent of ~200 μA was obtained at an incident power of 3.84 μW, and a photocurrent of ~180 μA was achieved under 3500 nm with 3.64 μW—both comparable to the photoresponse at 1550 nm. Consistent with the observations under 637 nm and 1550 nm, the *R* and EQE at both 2500 nm and 3500 nm exhibited a pronounced power-independent behavior (Figure 3e,f). Such power independence indicates that the device operates in the linear photoresponse regime, where the photocurrent scales linearly with the incident optical power. This behavior is in good agreement with a photobolometric mechanism: under the small temperature rise approximation, the temperature increase is proportional to the incident power, and thus the resulting change in resistance—and consequently the photocurrent—is also proportional to the power, yielding constant *R* and EQE across the measured power range.

**Figure 4:**
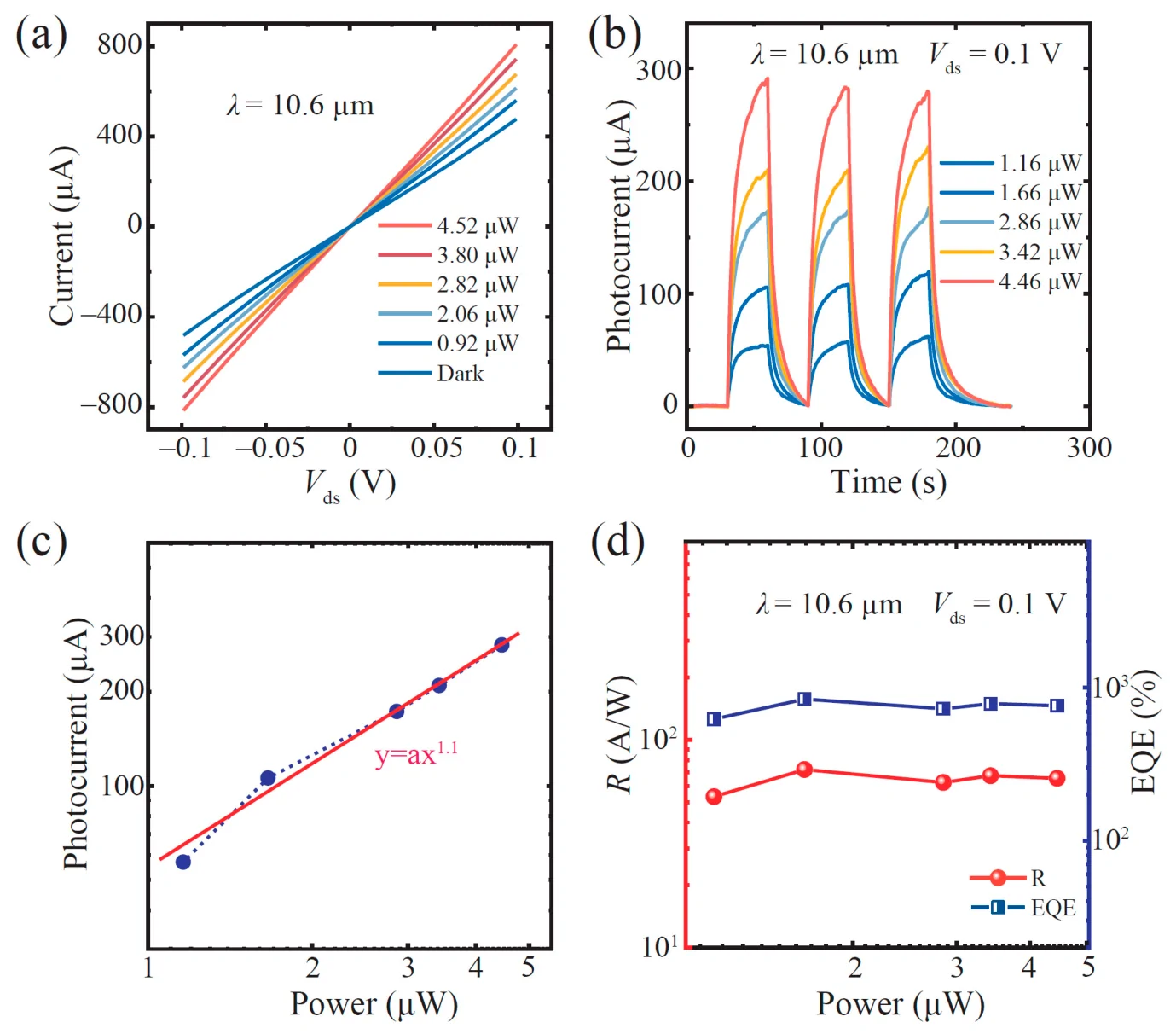
Optoelectronic response of the FePd_2_Te_2_ photodetector under 10.6 μm illumination. (**a**) *I*-*V* characteristics of the device under various incident powers. (**b**) Time-resolved photoresponse of the device under various incident powers. (**c**) Photocurrent as a function of incident power. (**d**) Extracted *R* and EQE of the device under various incident powers.

**Figure 5:**
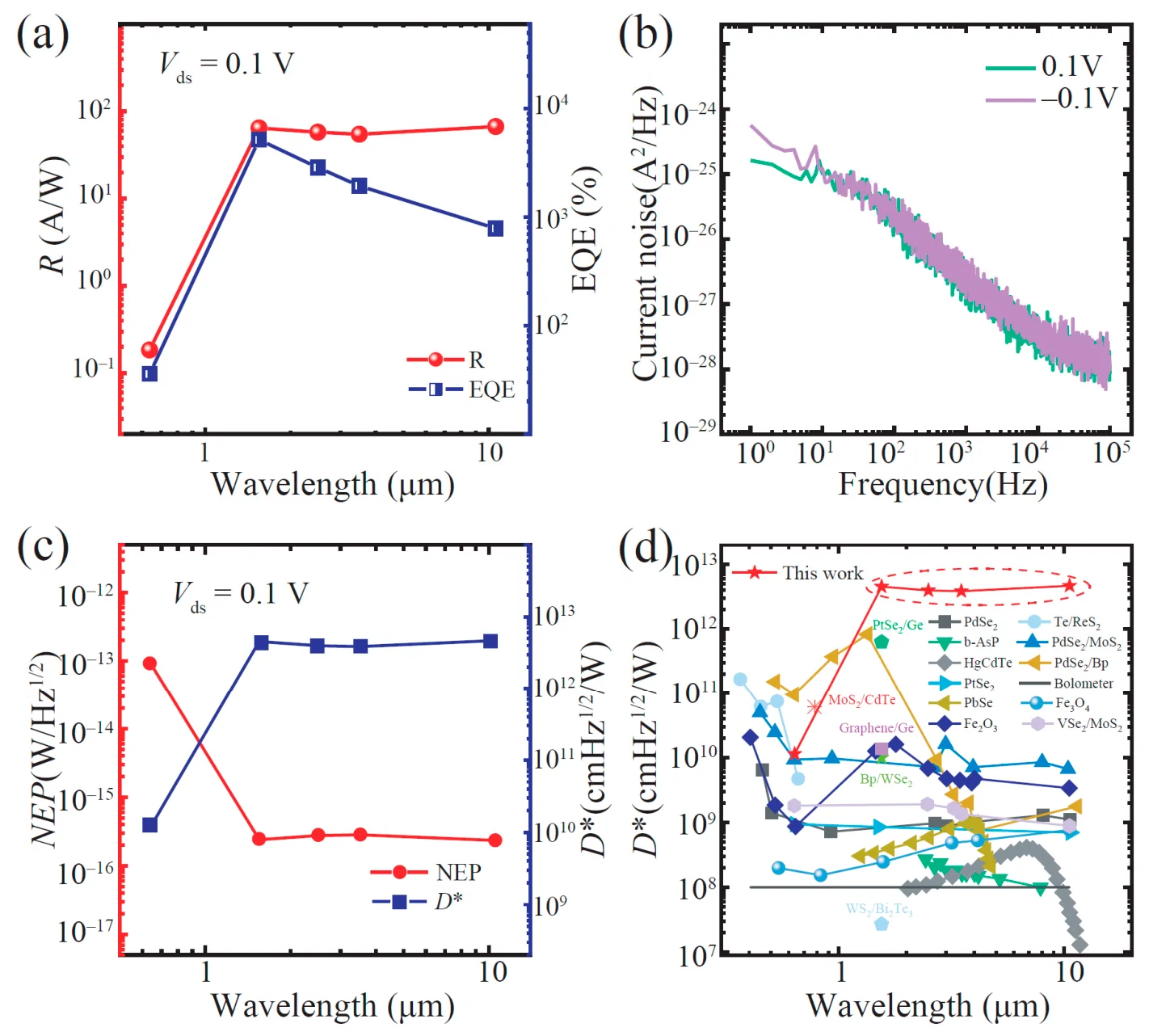
Comparison of the performance of the FePd_2_Te_2_ detector. (**a**) R and EQE of the device as functions of incident light wavelength at *V*_ds_ = 0.1 V. (**b**) Current noise power spectral of the device at different bias voltages. (**c**) *NEP* and D* of the device as a function of incident light wavelength at a bias of 0.1 V. (**d**) Comparison of D* versus the wavelength of the illumination for other 2D material-based photodetectors.

We further evaluated the device performance at 10.6 μm. The *I*-*V* curves and time-resolved photoresponse at 0.1 V are shown in Figure 4a and Figure 4b, respectively. The optoelectronic response at −0.1 V is shown in Supplementary Figure S2. A substantial photocurrent was obtained, comparable to those at shorter wavelengths. In addition, the photocurrent scales super-linearly with the measured incident optical power (Figure 4c), in contrast to the sublinear behavior observed at 637 nm illumination. This difference is likely attributed to the shallow absorption depth and fast surface heat dissipation, together with possible trap-assisted recombination at the contact interface at 637 nm, whereas at 10.6 μm the volumetric heating effect and the nonlinear temperature dependence of the contact resistance may dominate. As shown in Figure 4d, *R* and EQE exhibited power-independent behavior at 10.6 μm, consistent with the observations at shorter wavelengths. Figure 5a summarizes the *R* and EQE as a function of wavelength across the entire measured spectral range. Notably, the responsivity *R* remained nearly constant from 1550 nm to 10.6 μm, indicating a wavelength-independent photoresponse in the infrared regime. This is also consistent with the photobolometric model, where the photocurrent is primarily governed by the temperature rise at the contact interface rather than the photon energy. In contrast, the EQE showed a gradual decrease with increasing wavelength, from ~5 × 10^3^% at 1550 nm to ~1 × 10^3^% at 10.6 μm. This trend is a direct consequence of the definition EQE = *Rhc*/e*λ*: with *R* being approximately constant, the EQE scales inversely with *λ*, and thus decreases as the wavelength increases. It should be noted that in conventional semiconductor photodetectors, EQE represents the number of electron–hole pairs generated per incident photon, with an upper limit of unity in the ideal case. However, the value of ~5 × 10^3^% observed here does not signify that each incident photon generates 50 conduction electrons. Instead, it stems from the large current modulation efficiency of the photobolometric effect. That is, the temperature rise induced by photons modulates the device resistance, thereby producing an effectively amplified photocurrent signal. The combination of a wavelength-independent *R* and consistently high EQE across the entire 1550 nm–10.6 μm range highlights the excellent broadband performance of the FePd_2_Te_2_ EBL top-electrode photodetector, demonstrating its potential as a high-performance uncooled infrared photodetector.

Finally, we characterized the noise performance of the FePd_2_Te_2_ detector. Figure 5b shows the measured current noise power density spectra at ±0.1 V. The notably low noise currents demonstrate the device’s strong capability for detecting weak light signals. To quantify this, *NEP* and D* are employed to evaluate the sensitivity of the detector. *NEP* and D* can be calculated using the following formulas *NEP* = *i*_n_/*R*, and D* = *A*^1/2^ *R*/*i*_n_, where *A* is the effective area of the measured device. The noise current *i*_n_ can be obtained by calculating the mean-square-roots using the formula of ${<{i_{n}}^{2}>}^{1/2}=1/B\int_{0}^{B}S(f)df$, where *B* is the measured electrical bandwidth. Using the noise current and the measured responsivity *R* across the spectral range, we calculated the *NEP* and D* as functions of wavelength, as presented in Figure 5c. Remarkably, across the entire infrared range, the device exhibits an *NEP* below 2.83 × 10^−16^ W/Hz^1/2^ and a D* exceeding 3.86 × 10^12^ cm Hz^1/2^/W. Figure 5d summarizes D* as a function of wavelength for advanced 2D material detectors, which reflects the detection limit of the photodetectors. The FePd_2_Te_2_ device shows an outstanding D*, significantly superior to that of most 2D material devices across the entire infrared range. Owing to the high responsivity and low noise level, the device achieved excellent detection sensitivity. This indicates that FePd_2_Te_2_ has considerable application potential in the field of highly sensitive infrared detection.

## 4. Conclusions

In summary, we have demonstrated a room-temperature infrared photodetector based on FePd_2_Te_2_ nanoflakes that operates effectively from the visible to the long-wave infrared under a low bias of 0.1 V. The device exhibits a high responsivity, a low noise equivalent power, and an excellent specific detectivity across the entire measured infrared range. Through wavelength-dependent photoresponse analysis, the photoresponse is most consistent with a contact-resistance-dominated photobolometric effect, with conventional photovoltaic and photogating mechanisms unlikely to be the dominant origin. The pronounced response under NWIR and LWIR illumination is well explained by the increased optical penetration depth and the resulting volumetric heating at the contact interface, which produces a larger temperature rise and a stronger photocurrent. The combination of broadband operation, low bias, high sensitivity, and simple device architecture highlights the potential of FePd_2_Te_2_ for practical uncooled infrared detection. Future work should focus on optimizing contact engineering and device scaling to further improve response speed and integration compatibility, paving the way toward low-cost, high-performance infrared optoelectronic systems.

## Data Availability

The original contributions presented in this study are included in the article/Supplementary Materials. Further inquiries can be directed to the corresponding authors.

## Supplementary Materials

The following supporting information can be downloaded at: https://www.mdpi.com/article/10.3390/nano16201289/s1, Figure S1. Height profile of the FePd_2_Te_2_ flake; Figure S2. Optoelectronic response of the FePd_2_Te_2_ photodetector under various illumination at −0.1 V. (a–e) Time-resolved photoresponse of the device under various incident powers at 637 nm, 1550 nm, 2500 nm, 3500 nm, and 10.6 μm respectively. (f) The semilogarithmic plot of the *I*-*V* curves under 10.6 μm illumination; Table S1. Illumination conditions and optical-power calculation.
