# Supplementary material for: High-Performance Uncooled Infrared Photodetection Based on van der Waals Metallic FePd2Te2 Nanoflakes
Source: Nanomaterials (Basel). 2026 Oct 11;16(20):1289. doi: 10.3390/nano16201289 (PMC13707828; doi:10.3390/nano16201289)
Supplement: Supplementary file 1 [file nanomaterials-16-01289-s001.zip › nanomaterials-4613206-supplementary.pdf]

High-Performance Uncooled Infrared Photodetection Based on van der Waals Metallic $FePd_2Te_2$ Nanoflakes

Kang Zhang[1], Yeqi Shen[1], Wenke Xu[1], Tao Han[1], Yubing Tu[1]*, Suofu Wang[2]*

Mingsheng Long[1]

[1]State Key Laboratory of Opto-Electronic Information Acquisition and Protection Technology, Institutes of Physical Science and Information Technology, Anhui University, Hefei 230601, China

[2]School of Energy, Power and Safety, Anhui University of Applied Technology, Hefei, 230011, China.

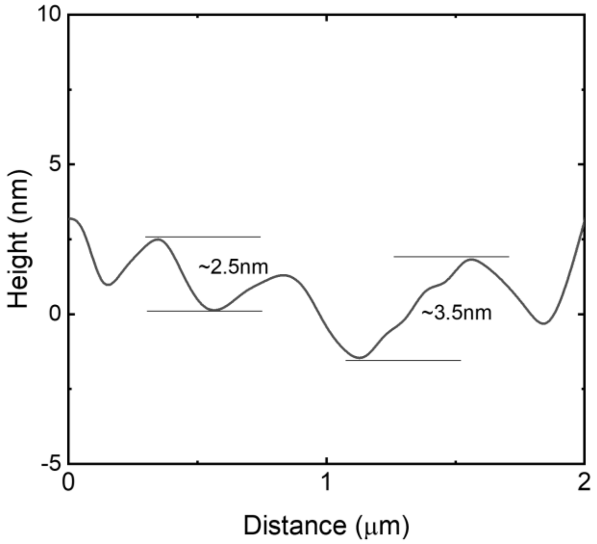

Figure S1. Height profile of the $FePd_2Te_2$ flake.

Table S1. Illumination conditions and optical-power calculation.

Wavelength	Diameter	Area	Power	Effective incident power
637 nm	60 μm	2826 μm^2	9.02/4.28/2.45 /0.92/0.13 μW	191.5/90.96/52.02 /19.62/2.72 μW
1550 nm	299.9 μm	70650 μm^2	3.34/2.99/1.84 /1.37/0.99 μW	2.84/2.54/1.56 /1.16/0.84 μW
2500 nm	4398.9 μm	15197600 μm^2	973/841/714 /527/344 μW	3.84/3.32/2.82 /2.08/1.36 μW
3500nm	4398.9 μm	15197600 μm^2	922/714/542 /426/223 μW	3.64/2.82/2.14 /1.68/0.88 μW
10.6 μm	4398.9 μm	15197600 μm^2	1130/866/724 /420/294 μW	4.46/3.42/2.86 /1.66/1.16 μW

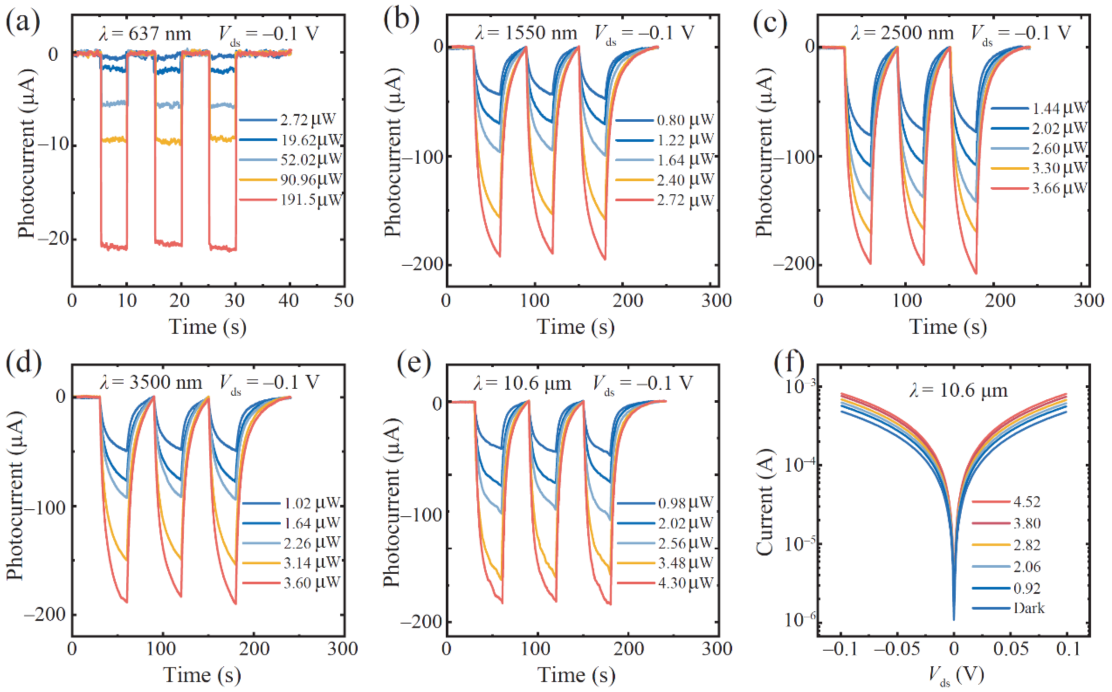

Figure S2. Optoelectronic response of the $FePd_2Te_2$ photodetector under various illumination at −0.1V. (a)–(e) Time-resolved photoresponse of the device under various incident powers at 637nm, 1550 nm, 2500 nm, 3500 nm, and 10.6 μm respectively. (f) the semilogarithmic plot of the I−V curves under 10.6 μm illumination.
